# Life-conditions and anthropometric variables as risk factors for oral health in children in Ladakh, a cross-sectional survey

**DOI:** 10.1186/s12903-021-01407-4

**Published:** 2021-02-05

**Authors:** Maria Grazia Cagetti, Fabio Cocco, Ezio Calzavara, Davide Augello, Phunchok Zangpoo, Guglielmo Campus

**Affiliations:** 1 Department of Biomedical, Surgical and Dental Science, University of Milan, Via Beldiletto 1, 20142 Milan, Italy; 2 Department of Surgery, Microsurgery and Medicine Sciences, School of Dentistry, University of Sassari, Viale San Pietro, 07100 Sassari, Italy; 3 Private Practitioner, Italian Association of Dentists (ANDI) Foundation Onlus, Via Giuseppe Ripamonti 44, 20141 Milan, Italy; 4 Dental Surgeon, Community Health Center, Padum, Zanskar, Ladakh, c/o Italian Association of Dentists (ANDI) Foundation Onlus, Via Giuseppe Ripamonti 44, 20141 Milan, Italy; 5 Department of Restorative, Preventive and Pediatric Dentistry, University of Bern, Freiburgstrasse 7, 3010 Bern, Switzerland; 6 School of Dentistry, Sechenov University, 119991 Moscow, Russia

**Keywords:** Oral health surveys, Dental caries, BMI, Waist circumference, Risk factors

## Abstract

**Background:**

The aim of this survey was to evaluate the severity of dental caries among children living in Zanskar Valley (Ladakh, India) and its association with anthropometric and background variables.

**Methods:**

This cross‐sectional survey was conducted on schoolchildren divided into four age groups (< 6, ≥ 6 < 11, ≥ 11 < 14 and > 14 years of age). A total of 1474 schoolchildren (607 males, 41.2%) were examined. Actual caries prevalence (dt/DT) and gingival bleeding were recorded by four calibrated dentists. An ad hoc questionnaire evaluated general health, eating habits, oral hygiene and the self-perception of oral conditions. Height, weight, waist circumference, heart-rate and oxygen-saturation were also collected directly by examiners. Responses to questionnaire items were treated as categorical or ordinal variables. The relationship between children’s caries data, gingival bleeding, gender, Body Mass Index (BMI) following the International Obesity Task Force, waist circumference and questionnaire items was assessed using the Kruskal–Wallis test and Pearson correlation. Conditional ordinal logistic regression was used to analyse associations among caries severity, gender, BMI, waist circumference, oxygen saturation and questionnaire items. A forward stepwise logistic regression procedure was also carried-out to estimate the ORs of gingival bleeding prevalence and the covariates derived from examination or questionnaire.

**Results:**

Caries was almost ubiquitarian with only 10.0% of caries-free children (dt/DT = 0). Caries severity, in both primary and permanent dentitions, was statistically significantly related to gender, waist circumference, BMI, oral hygiene frequency and self-reported chewing problems (*p* < 0.01 in both dentitions). An increasing relative risk for caries in permanent dentition compared to caries-free subjects was observed in children with a low BMI (RRR = 1.67, _95%_CI = 1.54/2.83 for subjects with 1–3 caries lesions and RRR = 1.52, _95%_CI = 1.36/1.74 for subjects with > 3 caries lesions); also, children with reduced waist circumference had a higher relative risk to have 1–3 caries lesions (RRR = 2.16, _95%_CI = 1.84/2.53) and an even higher risk to have more than 3 caries lesions (RRR = 4.22, _95%_CI = 3.33/5.34).

**Conclusions:**

A significant impact of untreated caries lesions was observed in Ladakh schoolchildren; low BMI values and reduced waist circumference showed to be the main caries risk predictors. Preventive and intervention programmes should be implemented to improve children's oral health.

## Background

Ladakh is an Indian region, comprising two districts: Leh, with a Buddhist majority, and Kargil, with a Muslim majority, and a population of around 280 thousand inhabitants. The Indian Government reports Ladakh to be one of the districts below standards in term of health services and conditions [1, 2]. The high altitude, above 9000 ft, is responsible for cold and an oxygen content equivalent to two thirds of that at sea level, limited diet, limited availability of drinking water with a low fluoride content (≤ 0.02 ppm/l) and poor socio-economic conditions [3–6]. Low obesity rates, physical growth patterns in height and arm circumference are reported [7–9].

Although great improvements have been made in global oral health, in the under-privileged populations such goal has not yet been achieved [10] and caries, in particular, is a health problem yet to be solved. Caries aetiology is a complex process with diet as one of its fundamental aetiological factors. Socio-environmental factors also play a strong role on oral health: they can produce damage to oral functions as well as affect the quality of life especially of disadvantaged groups of population. In developing countries, diet transition and inadequate exposure to fluorides are the main reasons for the growth of caries burden in these areas [11].

The Italian National Association of Dentists Foundation (ANDI) has among its main aims to improve the quality of life of populations with socio-economic inequalities in various parts of the world, as the population living in the Zanskar Valley of Ladakh. Dental care is limited to small dental clinic in Padum at the Community Health Center (CHC), the only hospital in the valley. During summertime, when the valley is accessible, teams of volunteers from ANDI Foundation work as dentists at the Zanskar Health Care & Sowa Rigpa Research Institute facility and promote oral health in schools.

At the best of Authors’ knowledge, no data regarding oral health of children living in Ladakh are available in literature. This epidemiological survey, in addition to filling this void, was conducted with the aim of collecting caries data in order to plan a preventive project to reduce caries through the use of Xylitol in children living in Ladakh.

The aim of the present survey was to assess the oral health conditions of a large sample of the children population aged from 5 to 17 years living in Zanskar Valley of Ladakh and to evaluate the association of oral variables with anthropometric measures, general health variables and life-style behaviours.

## Methods

The survey was planned as a cross-section evaluation. The study was conducted in Summer 2019. The study population comprised schoolchildren aged 5–17 years.

### Study area and population

The study was carried out from July to August 2019 in Zanskar Valley (Ladakh) in all the 18 schools (both public and private) located in and around the city of Padum, in some Buddhist monasteries and in some rural areas. Teachers from each involved school received a 2-h lesson on oral health prevention held in English, in the presence of a language mediator, in order for them to be able to teach this topic in class. The total schoolchildren population aged 5–17 years amounted to 2407 children. Due to language problems and the low literacy rate of the population, parents’ or guardians’ verbal approval was obtained for children’s participation; verbal consent was also obtained by children capable to give assent (eight years old children and older). The only inclusion criteria for eligibility was to be between 5 and 17 years old and to obtain the verbal consent to participate from parents/guardians or parents/guardian and child. No exclusions criteria were set. The study gained the approval of all the schools’ authorities and local health authority. The response rate was 100%.

### Data collection

Due to the large number of subjects to be examined, four dentists acted as raters. Before leaving Italy, the team received training consisting in one-day theoretical course, followed by another day of examination of extracted teeth. Finally, a clinical training was performed on 20 children, who were examined and re-examined after 72 h. Inter-examiner reliability, sensitivity, specificity, percentage agreement and kappa statistics were evaluated. Inter-examiner reliability ranged from 0.75 to 0.84 (K-Cohen) for sound and from 0.82 to 0.88 for caries lesions. Intra-examiner reliability ranged from 0.82 to 0.90 for sound teeth and from 0.84 to 0.91 for caries lesions. One examiner (EC) served as benchmark and conducted the theoretical and calibration sessions. During calibration, the team of examiners was also trained to measure and record the anthropometric indices. The Decayed–Missing–Filled (DMF and dmf) method proposed by WHO was used for caries lesion registration. The diagnostic threshold for the decayed tooth component (d/D) is the cavitated dentine lesion [12, 13]. Every subject was examined using: a plain mirror (Hahnenkratt, Königsbach, Germany) and the WHO CPI ballpoint probe (Asa-Dental, Milan, Italy), under standard light. When necessary, teeth were cleaned and/or dried using cotton rolls before the examination. No bitewing radiographs or fiber-optic trans-illumination were used. The presence of gingival bleeding after probing was evaluated only on six teeth [14].

An ad hoc questionnaire was build-up to assess general health, eating habits, oral hygiene and the self-perception of oral conditions, based on previous surveys (Additional file 1: Questionnaire) [15–18]. Direct face to face interviews were conducted with at least one parent of the children under the age of 12, while older children were asked to answer a self‐administered questionnaire. Ten items were related to dietary habits as the consumption of fresh fruit, biscuits, cakes, jam/honey, sweets/candy, meat, cheese, rice, chewing gum, soft drinks, sugared drinks; three items were related to hygiene habits as frequency of oral hygiene, use of a toothbrush and the perception of oral conditions.

The items related to general health (height, weight, waist circumference, heart-rate, oxygen saturation) were collected by the examiners as follows: the height (cm) was measured using a portable stadiometer (Seca 700, DE, Seca.com) asking the children to stand upright with the back (head, buttocks and heels) touching the device, wearing the school uniform and no cap or shoes were admitted; the weight (Kg) was also measured using a digital weight scale (Seca Clara 803, Seca, DE Seca.com); the waist circumference as the midway between lower ribs and the iliac crest was measured in cm after asking the children to stand with their arms wide open and feet positioned close together. The body mass index (BMI) was calculated dividing the weight by the height squared (kg/cm^2^); oxygen saturation and heart-rate were measured using a portable pulse oximetry device (Mindray PM-60, Mindray Medical Italy S.R.L).

The present study is reported according to the Reporting of Observational Studies in Epidemiology (Additional file 2: STROBE checklist) guidelines for cross-sectional studies [19].

### Statistical analysis

Data were entered into a database (Excel 2018; Microsoft Corporation, Redmond, WA, USA). Statistical analyses were performed using Stata® 16.0 software (http://www.stata.com). Responses to questionnaire items were treated as categorical or ordinal variables. The BMI data and waist circumference were then considered by the ethnicity of the population, re-coded using the International Obesity Task Force [20–23] and then categorized into three categories each, as thinness (BMI below or equal to 15), normal weight (BMI more then 15 or lower then 22) and overweight (BMI more than 22) and as reduced less (than 64 cm), normal (64–71 cm) and higher (> 71 cm) for waist circumference. Oxygen saturation data was divided in two classes under 90% and above 90% [24].

Mean caries data, BMI, waist circumference and oxygen saturation were calculated by age groups (< 6, ≥ 6 < 11, ≥ 11 < 14 and > 14 years of age). The relationship between children’s caries data, gingival bleeding, gender, BMI, waist circumference and questionnaire items were assessed using the Kruskal–Wallis test and a linear trend was calculated to aid the interpretation of data to determine if the measurements indicate an increasing or decreasing trend. Caries severity was evaluated separately for primary and permanent dentition following the distribution frequencies as follows: dt/DT = 0 (caries-free subjects), low caries severity dt in the range between 1 and 5 lesions, and DT in the range between 1 and 3 lesions, high caries severity dt > 5 and DT > 3 caries lesions [25, 26]. Conditional ordinal logistic regression was used to analyse associations among caries severity level (dt/DT) as dependent variable, gender, BMI, waist circumference, oxygen saturation and questionnaire items. The Akaike information criterion (AIC) was used to measure the goodness of fit of the statistical model. Multicollinearity might sometimes cause problems with regression results. This problem was solved using the DFBETA command in Stata, dropping the information that have too much influence on the regression line [18, 27].

A forward stepwise logistic regression procedure was also carried out to estimate the ORs of gingival bleeding prevalence and the covariates derived from clinical examination or questionnaire data.

## Results

Overall, 1474 children (61.2% of the population) were examined (Table 1), 607 males (41.2%) and 867 females (58.8%). All data are available as additional file (Additional 3: Raw data). The gender ratio was statistically different between living areas stratified by age group (χ^2^_(6)_ = 56.6 *p* < 0.01). Caries was almost ubiquitarian in the examined population with only 10.0% of children without caries lesions (dt/DT = 0) as is possible to read in Table 2. The highest mean number of caries lesions affecting primary dentition was recorded in younger groups (below 6 years of age and ≥ 6 < 11 years of age), as in permanent dentition the highest values were observed in the oldest age group. Caries severity, both in primary and permanent dentitions was statistically significantly related to gender, waist circumference, BMI, oral hygiene frequency and self-reported chewing problems (*p* < 0.01 in both dentition), while oxygen saturation was statistically significantly related to caries severity in permanent dentition (Tables 3 and 4). For these variables, significant linear trends (*p* < 0.05) of caries severity were found in all exposure categories.

**Table 1:** Sample distribution according to gender and living areas, stratified by age groups

Age groups	Gender	Living areas			Total
		Padum	Rural areas	Monastery	
		n (%)	n (%)	n (%)	n (%)
< 6 years	Males	77 (27.3)	45 (16.0)	2 (0.71)	124 (44.0)
	Females	84 (29.8)	48 (17.0)	26 (9.2)	158 (56.0)
≥ 6 < 11 years	Males	123 (25.6)	68 (14.1)	19 (4.0)	210 (43.7)
	Females	144 (29.9)	93 (19.3)	34 (7.1)	271 (56.3)
≥ 11 ≤ 14 years	Males	116 (26.0)	61 (13.7)	15 (3.4)	192 (43.0)
	Females	147 (32.9)	103 (23.0)	5 (1.1)	255 (57.1)
> 14 years	Males	56 (21.2)	18 (6.8)	7 (2.6)	81 (30.7)
	Females	150 (56.8)	32 (12.1)	1 (0.4)	183 (69.2)
	Total	897 (60.9)	468 (31.8)	109 (7.4)	1474 (100.0)

Males versus females by age-groups χ^2^_(3)_ = 14.72 *p* < 0.01

Males versus females by living areas χ^2^_(6)_ = 56.62 *p* < 0.01

**Table 2.** Descriptive valued of BMI, waist circumference adjusted by age, oxygen saturation, caries indices (dt/DT, mt/MT, ft/FT, dmft/DMFT) stratified by age-groups.

Mean value, Standard Deviation (SD)				
	< 6 years of age (n = 282)	≥ 6 < 11 years of age (n = 481)	≥ 11 ≤ 14 years of age (n = 447)	> 14 years of age (n = 264)
	Mean (SD)	Mean (SD)	Mean (SD)	Mean (SD)
BMI (IOTF)				
Thinness (≤ 15)	13.6 (1.9)	13.96 (1.4)	13.9 (0.9)	13.7 (0.5)
Normal weight (> 15 ≤ 22)	16.2 (1.1)	16.3 (1.3)	17.3 (1.6)	18.9 (1.6)
Overweight (> 22)	26.2 (5.5)	28.0 (5.1)	24.5 (1.3)	23.3 (1.5)
Waist circumference				
Reduced (< 64)	55.0 (3.9)	57.4 (3.5)	59.9 (4.0)	57.1 (6.5)
Normal (64–71)	66.1 (2.0)	66.6 (2.2)	67.2 (2.2)	68.5 (2.2)
Higher (< 71)	74.5 (2.4)	74.4 (2.4)	76.9 (4.2)	78.3 (4.3)
O_2_ saturation				
Under 90%	84.4 (5.8)	83.5 (7.2)	83.4 (12.1)	86.1 (3.2)
Above 90%	93.6 (2.5)	94.5 (2.6)	94.3 (3.0)	93.8 (2.7)
dt	6.4 (4.2)	5.0 (3.5)	0.9 (1.5)	0.1 (0.6)
DT	0.2 (0.6)	1.0 (1.2)	1.9 (2.3)	2.5 (2.4)
mt	– (–)	– (–)	– (–)	– (–)
MT	– (–)	– (–)	– (–)	0.69 (0.2)
ft	0.2 (0.1)	0.5 (0.14)	0.1 (0.3)	0.1 (0.5)
FT	– (–)	0.0.1 (0.3)	0.4 (0.7)	0.20 (1.1)
dmt	6.6 (4.4)	5.4 (3.7)	1.0 (1.8)	0.1 (0.8)
DMFT	0.2 (0.6)	1.1 (1.4)	2.3 (2.3)	3.3 (2.7)

**Table 3:** Sample distribution by gender, waist circumference adjusted by age, BMI, oral hygiene frequency and Self-reported chewing problem stratified by caries lesions categorization in primary dentition

Number of subjects = 916			
Gender	0 caries lesions	1–5 caries lesions	> 5 caries lesions
	n (%)	n (%)	n (%)
Males	132 (14.4)	130 (14.2)	115 (12.6)
Females	255 (27.8)	173 (18.9)	111 (12.1)

Waist circumference	0 caries lesions	1–5 caries lesions	> 5 caries lesions
	n (%)	n (%)	n (%)
Reduced	77 (8.4)	219 (23.9)	198 (21.6)
Normal	123 (13.4)	66 (7.2)	24 (2.6)
Higher	187 (20.4)	17 (1.9)	5 (0.6)

BMI (IOTF classification)	0 caries lesions	1–5 caries lesions	> 5 caries lesions
	n (%)	n (%)	n (%)
Thinness	108 (11.8)	113 (12.3)	105 (11.5)
Normal	268 (29.3)	175 (19.1)	109 (11.9)
Over weight	12 (1.3)	14 (1.5)	13 (1.4)

Oral hygiene frequency	0 caries lesions	1–5 caries lesions	> 5 caries lesions
	n (%)	n (%)	n (%)
Seldom	14 (1.5)	40 (4.8)	48 (5.2)
Less once a day	47 (5.1)	55 (6.0)	52 (5.7)
Once a day	218 (23.8)	135 (14.7)	86 (9.4)
Twice/day	109 (11.9)	72 (7.9)	40 (4.4)

Self-reported chewing problem	0 caries lesions	1–5 caries lesions	> 5 caries lesions
	n (%)	n (%)	n (%)
No	4 (0.4)	12 (1.3)	17 (1.9)
Seldom	108 (11.8)	87 (9.5)	65 (7.1)
Often	275 (30.0)	205 (22.4)	143 (15.6)

*χ*^2^_(1)_ linear trend = 27.07 *p* < 0.01

*χ*^2^_(2)_ linear trend 543.46 *p* < 0.01

*χ*^2^_(2)_ linear trend 43.70 *p* < 0.01

*χ*^2^_(3)_ linear trend 114.12 *p* < 0.01

*χ*^2^_(2)_ linear trend 28.36 *p* < 0.01

**Table 4:** Sample distribution by gender, waist circumference adjusted by age, BMI, oxygen saturation, sugared foods and drinks, oral hygiene frequency and Self-reported chewing problem stratified by caries lesions categorization in permanent dentition

Number of subjects = 1248			
Gender	0 caries lesions	1–3 caries lesions	> 3 caries lesions
	n (%)	n (%)	n (%)
Males	274 (22.0)	187 (15.0)	53 (4.3)
Females	264 (26.2)	307 (24.6)	101 (8.1)

Waist circumference	0 caries lesions	1–3 caries lesions	> 3 caries lesions
	n (%)	n (%)	n (%)
Reduced	429 (34.4)	212 (17.0)	31 (2.5)
Normal	93 (7.45)	150 (12.0)	47 (3.8)
Higher	77 (6.17)	132 (10.6)	75 (6.0)

BMI (IOTF classification)	0 caries lesions	1–3 caries lesions	> 3 caries lesions
	n (%)	n (%)	n (%)
Thinness	212 (17.0)	181 (14.5)	51 (4.1)
Normal	353 (28.3)	298 (23.9)	99 (7.9)
Over weight	36 (2.9)	14 (1.1)	3 (0.2)

Oxygen Saturation	0 caries lesions	1–3 caries lesions	> 3 caries lesions
	n (%)	n (%)	n (%)
≤ 90%	238 (19.1)	218 (17.5)	72 (5.8)
> 90%	362 (29.0)	277 (22.2)	81 (6.5)

Oral Hygiene Frequency	0 caries lesions	1–3 caries lesions	> 3 caries lesions
	n (%)	n (%)	n (%)
Seldom	88 (7.1)	41 (3.3)	9 (0.7)
Less once a day	110 (8.8)	86 (6.9)	14 (1.1)
Once a day	252 (20.2)	254 (20.3)	93 (7.5)
Twice/day	149 (11.9)	114 (9.1)	37 (3.0)

Sugared foods and drinks	0 caries lesions	1–3 caries lesions	> 3 caries lesions
	n (%)	n (%)	n (%)
Seldom	3 (0.24)	6 (0.48)	– (–)
Once a day	311 (24.9)	279 (22.4)	77 (6.2)
More than once a day	286 (22.9)	210 (16.8)	76 (6.1)

Self-reported chewing problem	0 caries lesions	1–3 caries lesions	> 3 caries lesions
	n (%)	n (%)	n (%)
No	30 (2.4)	14 (1.1)	1 (0.1)
Seldom	140 (11.2)	156 (12.5)	57 (4.6)
Often	430 (34.5)	324 (26.0)	95 (7.6)

*χ*^2^_(1)_ linear trend = 12.19 *p* < 0.01

*χ*^2^_(2)_ linear trend = 202.41 *p* < 0.01

*χ*^2^_(2)_ linear trend = 9.25 *p* < 0.01

*χ*^2^_(1)_ linear trend = 4.37 *p* = 0.04

*χ*^2^_(3)_ linear trend = 33.13 *p* < 0.01

*χ*^2^_(2)_ linear trend = 0.53 *p* = 0.75

*χ*^2^_(2)_ linear trend = 25.53 *p* < 0.01

The main dietary habits consisted of rice (95.5% of the sample reported to eat rice more than twice a day) and sugared drinks such as soft-drinks, *kholak*, sugared tea/coffee (96.9% of the sample reported more than twice a day). A statistically significant linear trend (χ^2^_(3)_ linear trend 34.2 *p* < 0.01) across the consumption of fruit was associated to caries severity (62.2% at least once a day) (*data not in table*).

Estimates related to caries severity in primary and permanent dentitions according to gender, waist circumference, BMI, oral hygiene frequency, self-reported chewing problems, oxygen saturation and sugared foods and drinks consumption (the latter two related to permanent dentition) are displayed in Table 5 without outliers. All the models were statistically significant (*p* < 0.01). An increasing relative risk for caries in permanent dentition compared to caries-free subjects was observed in children with a low BMI (RRR = 1.7, _95%_CI = 1.5/2.8 for subjects with 1–3 caries lesions and RRR = 1.5, _95%_CI = 1.4/1.7 for subjects with more than 3 caries lesions); children with reduced waist circumference also had a higher relative risk of having 1–3 caries lesions (RRR = 2.2, _95%_CI = 1.8/2.5) and an even higher risk of having more than 3 caries lesions (RRR = 4.2, _95%_CI = 3.3/5.3). The goodness of fit (AIC criterion) was 4012.7. Table 6 presents the OR estimates via the forward procedures in logistic regression by gingival bleeding. Subjects with reduced waist circumference were found to have twice the risk of gingival bleeding; also subjects with reduced oxygen saturation showed an increased risk of gingival bleeding (OR = 1.8 _95%_CI = 1.6/2.4).

**Table 5.** Multivariate analysis. Conditional multinomial regression between caries lesions categorization and background variables

In brackets is reported the reference of each background variable			
Primary dentition			
Number of observation = 916	χ^2^_(8)_ = 706.16	*p* < 0.01	log likelihood = − 1231.86
Base outcome caries-free subjects			

	Low caries severity			High caries severity		
	RRR (SE)	*p* >*|z|*	*95% CI*	RRR (SE)	*p* >*|z|*	*95% CI*
Gender (*male*)	0.8 (0.1)	0.12	0.6/1.1	**1.6 (0.2)**	** < 0.01**	**1.4/2.8**
Waist circumference (*reduced*)	**2.2 (0.1)**	** < 0.01**	**1.2/3.2**	**1.4 (0.1)**	** < 0.01**	**2.1/4.1**
BMI (*thinness*)	**1.3 (0.2)**	**0.03**	**1.0/1.8**	1.1 (0.2)	0.53	0.8/1.5
Oral Hygiene Frequency (*seldom*)	**1.8 (0.2)**	** < 0.01**	**1.6/2.9**	**1.6 (0.2)**	** < 0.01**	**1.5/3.2**

Permanent dentition			
*Number of observation* = *1248*	χ^*2*^_(*14*)_ = *240.22*	*p* < *0.01*	*log likelihood* = *-1318.35*
Base outcome caries-free subjects			

	Low caries severity			High caries severity		
	RRR (SE)	*p* >*|z|*	*95% CI*	RRR (SE)	*p* >*|z|*	*95% CI*
Gender (*male*)	1.2 (0.2)	0.09	1.0/1.5	1.3 (0.2)	0.21	0.9/1.8
Waist circumference (*reduced*)	**2.2 (0.2)**	** < 0.01**	**1.8/2.5**	**4.2 (0.5)**	** < 0.01**	**3.3/5.3**
BMI (*thinness*)	**1.7 (0.2)**	** < 0.01**	**1.5/2.8**	**1.5 (0.1)**	** < 0.01**	**1.4/1.7**
O2 saturation (≤ *90%*)	1.2 (0.1)	0.07	1.0/1.0	1.0 (0.1)	0.05	1.0/1.0
Oral Hygiene Frequency (*seldom*)	1.1 (0.1)	0.44	0.9/1.2	1.4 (0.1)	0.24	0.9/1.4
Sugared foods/drinks (*seldom*)	**1.8 (0.1)**	**0.01**	**1.6/1.9**	1.0 (0.2)	0.94	0.7/1.4
Self-reported chewing problem (*yes*)	0.8 (0.1)	0.08	0.7/1.0	1.1 (0.1)	0.05	1.0/1.2

The bold values represent the statistically significant results

**Table 6:** Multivariate analysis. Logistic regression between bleeding on probing and background variables

*In brackets is reported the reference of each background variable*	
*Number of observation* = *1474*	χ^*2*^_(*6*)_ = *24.90 p* < *0.01 log likelihood* = *-931.25*

Bleeding on probing	OR (SE)	*p* >*|z|*	*95% CI*
Waist circumference (*reduced*)	**2.2 (0.2)**	** < 0.01**	**1.8/2.5**
BMI (*underweight*)	0.9 (0.1)	0.07	0.7/1.0
O_2_ saturation (≤ *90%*)	**1.8 (0.2)**	**0.01**	**1.6/2.4**
Oral Hygiene Frequency (*seldom*)	1.1 (0.1)	0.27	0.1/1.2
Sugared foods/drinks (*seldom*)	1.2 (0.1)	0.09	0.1/1.5
Self-reported chewing problem (*yes*)	0.8 (0.1)	0.08	0.7/1.0

The bold values represent the statistically significant results

## Discussion

This is the first epidemiological survey carried out in an isolated valley of the remote region of Ladakh, which contributes to the characterization of oral health of a particular population of children living in an area where environmental conditions are extremely difficult. The oral conditions, weight and height, waist circumference, oxygen saturation, heart-rate and dietary and oral hygiene habits of about two thirds of the entire school population were examined at school.

For both dentitions, caries prevalence was very high in all age groups, for both genders and for different living areas. Caries experience was entirely due to decayed teeth, since fillings were few in all age groups.

A weak point of the survey could regard the caries index used (DMFT):it is not designed to record additional caries data such as the activity of the lesion or the extreme consequences of the caries process, as fistula or abscess [13]. On the other hand, the high number of children enrolled and the location of the examination (schools) calls for the use of an easy and quick method to record caries, such as the DMFT index.

There were more females than males in the sample; in the older group (> 14 years) males in particular, represented less than a third of the sample. Two different hypotheses can be made to explain this gender disparity: the early school leaving of males caused by their involvement in work activities, as reported in previous studies [29, 30], or their transfer to more prestigious schools outside the Valley, as families prefer to invest on the education of boys rather than girls [31].

Few data are available on caries in children population with similar background characteristics. While Nepalese schoolchildren showed a high caries prevalence in both dentitions with values lower than those recorded in Ladakh [32], the caries prevalence in Mongolian adolescents was higher, although the sample size was quite reduced [33]. Caries severity, as the number of dentine caries lesions for both dentitions, was associated to gender, waist circumference, BMI, oral hygiene frequency and self-reported chewing related problems. A rather large number of papers investigated the association between caries and anthropometric variables [33–35]. Children and adolescents living in high altitude areas present lower growth patterns than their counterparts living at sea level [6]. The Body Mass Index provides a convenient measure of a person’s fatness. The BMI proposed by the International Obesity Task Force (IOTF) was used to assess the prevalence of child overweight, obesity and thinness, as it provides more effective values of thinness especially in populations with a high prevalence of under-weight children [20]. No agreement is reported in literature on the relationship between BMI and dental caries due to the effect of confounders and effect modifiers [28]. Furthermore, in most of the studies, BMI was calculated according to the World Health Organization guidelines. Although evidence of the coexistence of obesity and dental caries has been reported, as the same risk factors are shared, a U-shaped relationship between severe dental caries and the two ends of anthropometric measures has been reported in Chinese children [29]. Dental caries was more common among low weight Indian children than among normal weight and overweight-obese children [30]. According to these findings, Ladakh children in the thinness BMI category and with a low waist circumference present a higher risk rate of caries than their peers with normal or high anthropometric variables. Waist circumference as well as BMI index, have shown to predict the clustering risk factor of cardiovascular disease and coronary artery disease [35, 36]. Since no longitudinal studies have examined the association between severe dental caries and both thinness and overweight, it is difficult to give reasons for their observed association. Overweight/obesity and thinness are different sides of the same coin: malnutrition. High intakes of saturated fat, sugared drinks and snacks, and refined carbohydrates intake produce overweight, whilst low intakes of foods rich in vitamins and proteins may cause thinness. This scarcity of highly nutritious foods is replaced by foods rich in sugars. In both cases, an inadequate intake of free-sugars may represent a risk for dental health, especially when protective caries factors are missing [37]. Waist circumference was also associated to gingival health: children with a reduced waist circumference show a double risk of bleeding on probing compared to normal or high values. However, BMI was not associated with bleeding, making the association between gingival bleeding and malnutrition unclear. A limited diet, low in fresh fruit and vegetables, is a factor influencing malnutrition, especially with regard to dietary intake of vitamins. In a recent metanalysis investigating the role of vitamins on oral health, vitamin C deficiency was found to be associated with gum bleeding; a reduction of the gingival problem was recorded after vitamin supplementation [38]. Obesity has been linked to increased inflammatory activity in gingival fluid [36] and obese children with Type 2 diabetes show a trend towards poorer oral health compared to normal weight and obese children without diabetes [39]. As far as the Authors know, this is the first paper that found an association between gingival health and low anthropometric values.

Poor oral hygiene habits are recognized as a risk factor both for caries and gingivitis in children and adults [14, 16, 40, 41]. In childhood, behavioural changes in relation to hygiene habits can have an important influence on the oral health status of future adults [40]. About a quarter of the children in Zanskar valley of Ladakh, with both primary and permanent dentitions, brush their teeth less frequently than once a day; this poor habit increases the risk for caries, especially in younger children. Both pre-school children and adolescents who brushed their teeth more than once daily showed less caries experience than those who brushed their teeth once per day [42, 43]. These outcomes could be related to several factors among which the poor knowledge of hygienic standards and the lacking availability of many imported consumer products, such as toothbrushes and toothpastes.

## Conclusions

This survey highlights to what extent oral health can be considered a burdensome issue in the children population of a remote area of India. Anthropometric variables linked to malnutrition (i.e. low BMI and reduced waist circumference) and poor oral hygiene habits were associated with a high prevalence of untreated dental caries leading to frequent chewing problems. Effective preventive programmes need to be implemented at school-level to improve oral health of children living in Ladakh.

## Supplementary Information

**Additional file 1.** Questionnaire.

**Additional file 2.** STROBE Checklist.

**Additional file 3.** Raw data.

## Data Availability

All data generated or analysed during this study are included in this published article and/or in additional files. The data were available as Additional file 3: Raw data.

## Abbreviations

dmft: Decayed, missed, filled teeth (primary dentition)
DMFT: Decayed, missed, filled teeth (permanent dentition)
CPI: Community Periodontal Index
RRR: Relative risk ratio
95%CI: 95% Confidence interval
BMI: Body Mass Index
IOTF: International obesity task force
WHO: World Health Organization
ORs: Odds ratio
