# Supplementary material for: Life-conditions and anthropometric variables as risk factors for oral health in children in Ladakh, a cross-sectional survey
Source: BMC Oral Health. 2021 Feb 5;21:51. doi: 10.1186/s12903-021-01407-4 (PMC7863482; doi:10.1186/s12903-021-01407-4)
Supplement: Supplementary file 1 — Additional file 1. Questionnaire. [file 12903_2021_1407_MOESM1_ESM.pdf]

Oral Health Surveys

Basic Methods

5th Edition

World Health Organization

World Health Organization

Oral Health Questionnaire for Children

First, we would like you to answer some questions concerning yourself and your teeth

Identification number	Sex		Location		
	Boy	Girl	Urban	Periurban	Rural
1. ☐☐☐☐	☐	☐	☐	☐	☐
1 4	1	2	1	2	3

2. **How old are you today?** ____________________
(Years)

3. **How would you describe the health of your teeth and gums?**
(Read each item)

	Teeth	Gums
Excellent	☐ 1	☐ 1
Very good	☐ 2	☐ 2
Good	☐ 3	☐ 3
Average	☐ 4	☐ 4
Poor	☐ 5	☐ 5
Very poor	☐ 6	☐ 6
Don't know	☐ 9	☐ 9

4. **How often during the past 12 months did you have toothache or feel discomfort due to your teeth?**

Often	☐ 1
Occasionally	☐ 2
Rarely	☐ 3
Never	☐ 4
Don't know	☐ 9

Now please answer some questions about the care of your teeth

5. **How often did you go to the dentist during the past 12 months?**
(Put a tick/cross in one only)

Once	☐ 1
Twice	☐ 2
Three times	☐ 3
Four times	☐ 4

More than four times .... ☐ 5
I had no visit to dentist during the past 12 months .... ☐ 6
I have never received dental care/visited a dentist .... ☐ 7
I don't know/don't remember .... ☐ 9

If you did not see a dentist during the last 12 months, go on to question 7

6. **What was the reason for your last visit to the dentist?**
(Put a tick/cross in one box only)
Pain or trouble with teeth, gums or mouth .... ☐ 1
Treatment/follow-up treatment .... ☐ 2
Routine check-up of teeth/treatment .... ☐ 3
I don't know/don't remember .... ☐ 9

7. **How often do you clean your teeth?**
(Put a tick/cross in one box only)
Never .... ☐ 1
Several times a month (2–3 times) .... ☐ 2
Once a week .... ☐ 3
Several times a week (2–6 times) .... ☐ 4
Once a day .... ☐ 5
2 or more times a day .... ☐ 6

8. **Do you use any of the following to clean your teeth or gums?**
(Read each item)

	Yes 1	No 2
Toothbrush	☐	☐
Wooden toothpicks	☐	☐
Plastic toothpicks	☐	☐
Thread (dental floss)	☐	☐
Charcoal	☐	☐
Chewstick/miswak	☐	☐
Other	☐	☐

Please specify ____________________

9.
a) **Do you use toothpaste to clean your teeth** .... Yes ☐ 1 No ☐ 2
b) **Do you use toothpaste that contains fluoride?** .... Yes ☐ 1 No ☐ 2
Don't know .... ☐ 9

10. **Because of the state of your teeth and mouth, have you experienced any of the following problems during the past year?**

	Yes 1	No 2	Don't know 0
(a) I am not satisfied with the appearance of my teeth	☐	☐	☐
(b) I often avoid smiling and laughing because of my teeth	☐	☐	☐
(c) Other children make fun of my teeth	☐	☐	☐
(d) Toothache or discomfort caused by my teeth forced me to miss classes at school or miss school for whole days	☐	☐	☐
(e) I have difficulty biting hard foods	☐	☐	☐
(f) I have difficulty in chewing	☐	☐	☐

11. **How often do you eat or drink any of the following foods, even in small quantities?**
(Read each item)

	Several times a day 6	Every day 5	Several times a week 4	Once a week 3	Several times a month 2	Never 1
Fresh fruit	☐	☐	☐	☐	☐	☐
Biscuits, cakes, cream cakes, sweet pies, buns etc	☐	☐	☐	☐	☐	☐
Lemonade, Coca Cola or other soft drinks	☐	☐	☐	☐	☐	☐
Jam/honey	☐	☐	☐	☐	☐	☐
Chewing gum containing sugar	☐	☐	☐	☐	☐	☐
Sweets/candy	☐	☐	☐	☐	☐	☐
Milk with sugar	☐	☐	☐	☐	☐	☐
Tea with sugar	☐	☐	☐	☐	☐	☐
Coffee with sugar	☐	☐	☐	☐	☐	☐

(Insert country-specific items)

12. **How often do you use any of the following types of tobacco?**
(Read each item)

	Every day	Several times a week	Once a week	Several times a month	Seldom	Never
	6	5	4	3	2	1
Cigarettes, pipe or cigars ...	☐	☐	☐	☐	☐	☐
Chewing tobacco or snuff ..	☐	☐	☐	☐	☐	☐

13. **What level of education has your father completed (or your stepfather, guardian or other male adult living with you)?**

No formal schooling.. ☐ 1
Less than primary school.. ☐ 2
Primary school completed .... ☐ 3
Secondary school completed.. ☐ 4
High school completed .... ☐ 5
College/university completed .... ☐ 6
No male adult in household .... ☐ 7
Don't know... ☐ 9

14. **What level of education has your mother completed?**

No formal schooling.. ☐ 1
Less than primary school.. ☐ 2
Primary school completed .... ☐ 3
Secondary school completed.. ☐ 4
High school completed .... ☐ 5
College/university completed .... ☐ 6
No female adult in household... ☐ 7
Don't know... ☐ 9
(Insert country-specific categories)

That completes our questionnaire
Thank you very much for your cooperation!

Year	Month	Day	Interviewer	District	Country
☐☐	☐☐	☐☐	☐☐	☐☐	☐☐☐☐
